# Macaques preferentially attend to intermediately surprising information

**DOI:** 10.1098/rsbl.2022.0144

**Published:** 2022-07-06

**Authors:** Shengyi Wu, Tommy Blanchard, Emily Meschke, Richard N. Aslin, Benjamin Y. Hayden, Celeste Kidd

**Affiliations:** ^1^ Department of Psychology, University of California, Berkeley, 2121 Berkeley Way West, Berkeley, CA 94720, USA; ^2^ Klaviyo, 125 Summer St, Floor 6, Boston, MA 02111, USA; ^3^ Helen Wills Neuroscience Institute, University of California, Berkeley, 175 Li Ka Shing Center, MC 3370, Berkeley, CA 94720, USA; ^4^ Haskins Laboratories, Yale University, 300 George Street, New Haven, CT 06511, USA; ^5^ Department of Neuroscience, University of Minnesota, 321 Church St SE, Minneapolis, MN 55455, USA

**Keywords:** attention, statistical learning, eye tracking, rhesus macaque

## Abstract

Normative learning theories dictate that we should preferentially attend to informative sources, but only up to the point that our limited learning systems can process their content. Humans, including infants, show this predicted strategic deployment of attention. Here, we demonstrate that rhesus monkeys, much like humans, attend to events of moderate surprisingness over both more and less surprising events. They do this in the absence of any specific goal or contingent reward, indicating that the behavioural pattern is spontaneous. We suggest this U-shaped attentional preference represents an evolutionarily preserved strategy for guiding intelligent organisms toward material that is maximally useful for learning.

## 1. Introduction

Intelligent organisms acquire knowledge through experience; however, there is more information available than they can actually explore [1,2]. Thus, intelligent organisms must be selective.

Adaptive theories of curiosity posit that uncertainty helps guide learners’ exploration [3–9]. Specifically, adaptive learners attend to information of intermediate uncertainty. This results in a U-shaped relationship between uncertainty and inattention: low uncertainty events offer little to learn from and high uncertainty events are beyond the learners' processing capabilities [3,8,10–18]. This mechanism has been attested in humans and may represent an elegant solution for intelligent organisms to resolve the information overload problem.

Human infants and children preferentially maintain attention to sequential events of intermediate surprisal values [16–19]. While this pattern has not been observed in non-humans, monkeys can seek information for its inherent value. Rhesus macaques' inferotemporal cortical neurons respond more strongly to images presented in an unexpected order [20,21]. Further, macaques' behaviour demonstrates they will sacrifice liquid reward in exchange for information with no strategic benefit [22,23] and engage in directed exploration [24,25]. These data raise the possibility that strategic information-seeking patterns may reflect an evolutionarily ancient capacity for adaptive regulation of incoming information. If so, this would demonstrate a general principle of advanced evolved learners rather than a uniquely human skill.

Here, we employ a variation on the infant paradigm with rhesus macaques. We test the hypothesis that adaptive regulation of information-seeking is a cognitive skill shared with our common ancestor. Unlike most previous work on curiosity in macaques, we employ a free-viewing paradigm without rewards tied to particular responses. This approach tests for spontaneous preference and avoids possible learning effects. We find that macaques' visual attention is strikingly similar to that of human infants.

## 2. Methods

### (a) Subjects

Five male rhesus macaques (*Macaca mulatta*) served as subjects. Subjects had been trained to perform oculomotor tasks for liquid rewards through positive-reward-only reinforcement training using standardized methods [26] (electronic supplementary material, appendix S2).

### (b) Stimuli

**Figure 1.**
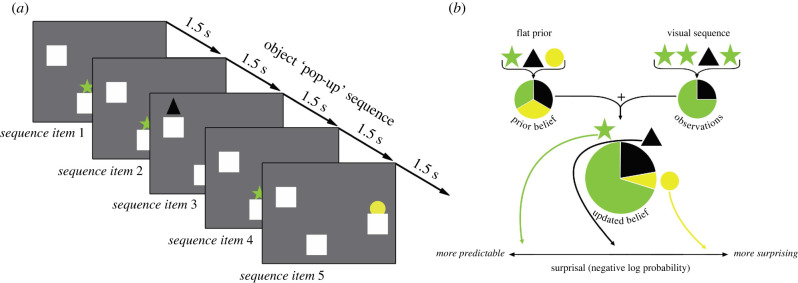
(*a*) Sequential visual display. The illustration shows five time-points in the sequence. At each event in the sequence, one of the three unique objects popped up from behind one of three boxes. (*b*) Idealized learning model schematic. The schematic shows how the idealized learning model forms probabilistic expectations about the expectedness of the next event in a sequence.

Visual stimuli were coloured shapes on a computer monitor (figure 1*a*). We designed the displayed stimuli to be easily captured by a simple statistical model [16,18]. Each trial featured one of 80 possible visual-event sequences (electronic supplementary material, appendix S1). All sequences were presented to all subjects in different randomized orders. One sequence was presented per trial, and each was presented in the form of a unique animated display.

Each animated display featured three identical boxes in three distinct, randomly chosen spatial locations that remained static throughout the sequence on the screen. Each box concealed one unique geometric object, which was randomly selected from a set that included four different shapes in eight colours (e.g. a yellow triangle and a blue circle). Geometric objects remained associated with their respective boxes throughout the sequence and were unique within a trial, but were chosen randomly from the set across trials [16–19].

Objects appeared from boxes on the displays according to the sequence orders. Each *event* within a sequence consisted of one of the three objects popping out from behind one of the three boxes (750 ms), and then back into the box (750 ms) without overlap or delay. Eighty sequences were generated to maximize the difference of their theoretical information property, such that the pop-up probabilities of each geometric object varied if a different sequence was observed. For example, if a sequence starts with 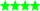 and follows by another 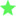, this is an example of a very predictable sequential event. If the same sequence starts again with 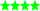 but follows by 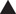, this would be an example of a less predictable sequential event.

### (c) Procedure

We recorded eye movements as subjects watched sequential visual displays designed to elicit probabilistic expectations, following methods employed in preverbal infants [16] and gaze-based experimental protocols for studying animal visual perception and cognition [27]. Eye positions were measured with the Eyelink Toolbox and were sampled at 1000 Hz by an infrared eye-monitoring camera system (SR Research, Osgoode, ON, Canada) [28]. A solenoid valve delivered a 53 µl water reward when each object was at its peak (every 1.5 s), regardless of where or whether the subject was looking. The intermittent and fully predictable reward is a standard procedure in primate behaviour studies designed to increase general task participation without making any particular task events reward associated [26]. Regardless of subjects' gaze behaviour, each sequence was displayed in full. The rate of presentation was between 0 and 2 trials, interspersed within unrelated trials for other studies [26,29,30].

### (d) Analysis

We analysed three behavioural measures: reaction time (RT), predictive-looking, and look-away. *RT* measures the latency to shift gaze to the object after it appears. This is a standard measure to detect agents' expectations. *Predictive-looking*^1^ is a binary variable that indicates whether the subject was already looking at the current object when it first became active but before the object actually popped up. *Look-away*^2^ is the first point in the trial when the macaque looked off-screen for 0.75 s (50% of the total pop-up event duration) [16–18]. We analysed these three behavioural measures as a function of the surprisal value of each event in the sequence, which is the negative log probability of the event's occurrence, according to unigram and transitional (or bigram) Markov Dirichlet-multinomial (ideal observer) models (following the analysis methods of [16–18]). The unigram model treats each event as statistically independent, while the transitional model assumes event order dependence and tracks the conditional probability on the immediately preceding event (electronic supplementary material, appendix S3). The models begin with an uninformative prior corresponding to the implicit beliefs a learner possesses before making any observations. Once the sequence presentation begins, the model estimates the surprisal value of the current event at each item in the sequence. It combines the simple prior with the learner's previous observations from the sequence in order to form a posterior or updated belief. The next object pop-up event then conveys the surprisal value according to the probabilistic expectations of the updated belief (figure 1*b*). We evaluated the statistical significance of variables using mixed effect linear and logistic regressions with random intercepts. The raw regression models include standardized linear and quadratic surprisal terms as predictors. The controlled regression models include covariate factors, such as whether an object is a repeat, distance between the current and previous pop-up object, trial number. A generalized additive model (GAM) was used to visualize the relationship between the surprisal estimate from the computational model and the behavioural data [31] (electronic supplementary material, appendix S3).

## 3. Results

### (a) Quicker deployment of gaze for events of intermediate surprisal

**Figure 2.**
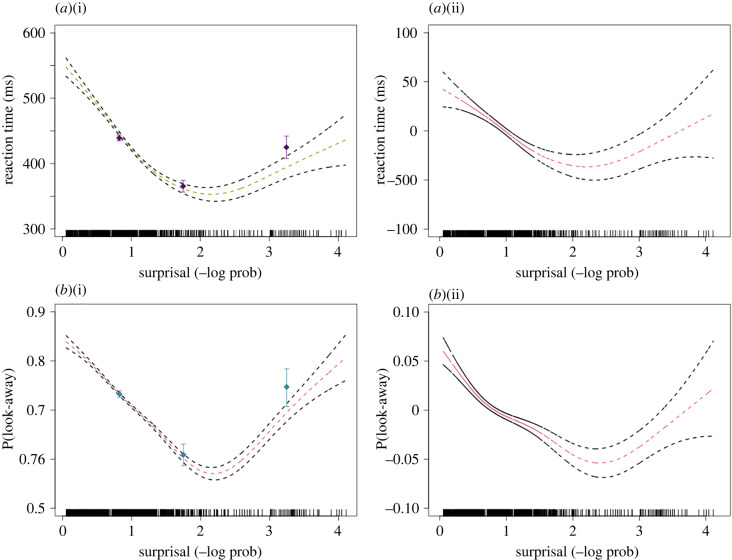
(*a*) RT (ms) as a function of unigram surprisal. (i) Subjects' RT to fixate the active object (*y*-axis) as a function of unigram surprisal (*x*-axis). The points and error bars show raw data binned to three group means of three evenly spaced intervals according to surprisal values. The smooth curve shows the fit of a GAM with standard errors. Vertical tick marks show values of surprisal attained in the experiment. (ii) RT (*y*-axis) and unigram surprisal (*x*-axis), while controlling for all factors. (*b*) Look-away probability as a function of unigram surprisal. (i) Subjects' probability of looking away (*y*-axis) as a function of unigram surprisal (*x*-axis). The smooth curve shows the fit of a GAM with 95% confidence interval. (ii) The relationship between look-away probability (*y*-axis) and unigram surprisal (*x*-axis), while controlling for all covariate factors.

The unigram GAM analysis shows that the relationship between RTs and subjects' expectations about stimulus predictability is U-shaped, with subjects exhibiting the fastest RTs for intermediately predictable stimuli (figure 2*a*(i)). We fitted the model with a log-transformed RT variable to ensure assumptions of linear mixed effect regression are fulfilled. The raw regression reveals both a significant linear term (*β* = −4.48, *t* = –5.58, *p* = 2.48 × 10^−8^) and a significant quadratic term (*β* = 5.43, *t* = 6.77, *p* = 1.42 × 10^−11^). The U-shape relationship holds when other variables are controlled in the GAM, as well as revealed by the significant quadratic term (*β* = 2.40, *t* = 2.55, *p* = 0.011) in the controlled regression (figure 2*a*(ii)). The significance of the quadratic term likely corresponds to a genuine U over the range of surprisal, especially in light of the fact that the significance holds even in the controlled GAM. In the GAM analysis for the transitional surprisal measures, it shows a shallower U-shape, with both linear trend (*β* = −2.54, *t* = −3.18, *p* = 0.001) and quadratic trend (*β* = 2.57, *t* = 3.21, *p* = 0.001) being significant in the raw model. Once all predictors are included, the curve becomes mostly flat. This shows that the unigram model is more robust than the transitional model to capture the relationship between subjects' RTs and the surprisingness of stimuli. Our results also show that all five subjects exhibit similar preference for stimuli of intermediate surprisal, suggesting that the U-shape relationship holds within rhesus macaques and is not due to subject average (electronic supplementary material, appendix S7). This consistent pattern observed in each macaque subject was also found within individual human infants who reserve attention for events that are moderately predictable [19] (electronic supplementary material, appendix S5 and S6).

### (b) Predictive looks towards unshown items

Subjects are more likely to predictively look at objects on their first appearance when the pop-up events are estimated to be more likely, according to the model. The GAM plot shows a decreasing trend between the probability of predictive-looking and the surprisal value (electronic supplementary material, appendix S2). The pattern is supported by the statistically significant linear surprisal terms in both unigram raw regression (*β* = −13.53, *z* = −3.02, *p* = 0.003) and the bigram raw regression (*β* = −12.85, *z* = −2.74, *p* = 0.006). This decreasing linear trend also holds in controlled models with linear terms being significant in unigram (*β* = −12.68, *z* = −2.3, *p* = 0.02) and transitional (*β* = −10.12, *z* = −1.98, *p* = 0.048) models. These results show that subjects might be curious about unknown information and spontaneously track the incoming statistics, expecting that there is some change that will occur and, if it does, it will be informative. They also suggest that as it is increasingly unlikely to see unopened boxes ever open, macaques are less likely to allocate their attentional resources towards monitoring unopened boxes. It is further evidence that macaques' information-seeking is moderated by their expectation in the absence of overt rewards (electronic supplementary material, appendix S5 and S6).

### (c) Preferential gaze towards events of intermediate surprisal

Estimated by the unigram GAM analysis, subjects were more likely to terminate attention to highly predictable events and also highly unexpected events (figure 2*b*(i)). In the raw regression, both the linear term (*β* = −34.66, *z* = −15.00, *p* < 2 × 10^−16^) and the quadratic term (*β* = 29.01, *z* = 11.68, *p* < 2 × 10^−16^) are statistically significant. The controlled logistic regression revealed statistically significant linear (*β* = −16.12, *z* = −4.73, *p* = 2.30 × 10^−6^) and quadratic terms (*β* = 6.38, *z* = 2.02, *p* = 0.04). Results from the transitional model show that there is a U-shaped relationship in the raw model and model fits, with the quadratic trend being statistically significant (*β* = 14.49, *z* = 6.72, *p* = 1.78 × 10^−11^). However, this pattern disappears when other variables are controlled (electronic supplementary material, appendix S5 and S6).

## 4. Discussion

Humans do not indiscriminately absorb any information they encounter. Instead, we preferentially seek out information that is maximally useful [1,2,8,32]. This regulated information-gathering strategy favours moderately surprising events, resulting in an inverse-U-shaped pattern between event surprisal and engagement. Here we show that this pattern, previously only observed in humans [16–19], is also observed in rhesus macaques, a primate species that diverged from humans roughly 25 million years ago.

The presence of this pattern in macaques suggests that the capacity to adaptively seek useful information is not uniquely human, but instead reflects long-standing evolutionary pressures present since at least the time of our last common ancestor. This is important because a good deal of theorizing highlights the uniqueness of human curiosity, with the implication that curiosity is a factor that has driven human divergence [33]. Our results, then, suggest an alternative hypothesis that humans and animals share a broad suite of cognitive adaptations. We suggest that these kinds of cognitive adaptations can flexibly adapt a primate's probabilistic beliefs to changing environmental statistics in order to implicitly guide learning in a broad range of domains, from learning about objects [34] to the social world [35,36]. Monkeys differed from humans in that unigram statistics were more robust predictors of monkey learners' behaviours than the transitional statistics to sequential stimuli [16,17]. While it may be tempting to wonder whether this reflects a species-level difference, this conclusion is premature and unlikely for several reasons. First, the macaques we tested had substantial experiences with tasks for which tracking unigram statistics was more relevant (e.g. k-arm bandit tasks) [22,25,28,30]. Second, previous work has demonstrated that macaques possess sensitivity to transitional statistics in other tasks [20,21,37–39]. Thus, further work with a macaque population with more similar experiences to the human infants would be required to draw strong conclusions about cross-species differences in unigram versus transitional statistical sensitivities.

Letting uncertainty guide attention is a broadly useful organizing principle for learning, including information relevant to avoiding predation and generating social expectations including those relating to mating. We suspect that this is likely a feature of intelligent organisms in general since its utility is not limited to especially social species. Though we have presented evidence that uncertainty drives attention in both humans and macaques, we note that uncertainty is not the only driver of attention. Perceptual salience (e.g. contrast, movement, colour saturation, visual complexity), social relevance (e.g. faces) and social pressures (e.g. dominance hierarchies, social familiarity) also guide attention [40–42]. Further work will be needed to determine to what degree each of these pertinent attentional drivers relatively influences attention across species, and how species-specific needs and pressures might influence their relative importance.

## Data Availability

All data and code can be found at https://doi.org/10.6078/D15Q7Q.

## Endnotes

1 In this paradigm, the events occurred temporally predictable (every 1500 ms), with no breaks between. Thus, these predictive looks differ from those elicited in most predictive-looking paradigms, where delays between events specifically encourage predictive looking.

2 In this version, sequences kept unfolding no matter whether the macaques were looking. This is different from the human infant paradigm, in which infants could terminate the displays with their inattention.
